# Impact of physical activity and BMI on health-related quality of life in overweight Indian adolescents: A randomized controlled study

**DOI:** 10.1016/j.obpill.2025.100201

**Published:** 2025-08-16

**Authors:** Prateek Srivastav, K. Vaishali, H. Vinod Bhat, Suzanne Broadbent

**Affiliations:** a Manipal College of Health Professions Manipal Department of Physiotherapy, Manipal, Karnataka, India; b Manipal College of Health Professions Department of Physiotherapy at Manipal, Manipal, Karnataka, India; c The Apollo University, Andhra Pradesh, India; d University of the Sunshine Coast, Queensland, Australia

**Keywords:** Health-related quality of life, Adolescents, Obesity, Physical activity, BMI, India, Randomized controlled trial

## Abstract

**Background:**

Health-related quality of life (HRQOL) is a critical yet often overlooked component of adolescent obesity management. While physical activity (PA) and body mass index (BMI) are established markers of health, their combined influence on HRQOL in Indian adolescents remains underexplored.

**Objective:**

To examine how PA and BMI influence overall and domain-specific HRQOL in overweight and adolescents with obesity following a structured lifestyle program.

**Methods:**

In this randomized controlled study, 604 adolescents (aged 11–16 years) with BMI above the 85th percentile were recruited from schools in Udupi, India. Individuals were randomized into three groups: multifactorial strategy (MFI: exercise, lifestyle education, behavioral counseling), exercise-only (EX), and Control group (CON). PA was measured using the Physical Activity Questionnaire for Adolescents (PAQ-A), and HRQOL was measured via the Pediatric Quality of Life Inventory (PedsQL 4.0). Linear mixed-effects models evaluated associations between PA, BMI, and HRQOL over a 12-month period, which included a 12-week intervention followed by 9 months of follow-up.

**Results:**

PA was linked with better HRQOL in the MFI (β = 9.30, p < 0.001) and EX (β = 4.47, p < 0.001) groups. Interestingly, higher BMI correlated with better HRQOL scores in both program arms. The CON group showed no meaningful association between PA and HRQOL. The domain-level analysis found that PA and BMI were positively linked to physical, emotional, social, and school functioning in both program groups, with more pronounced effects in the MFI group. The CON group showed improvement only in emotional functioning related to BMI.

**Conclusion:**

Structured PA programs, especially those combining education and behavioral support, notably enhance the quality of life among overweight Indian adolescents. These findings reinforce the importance of focusing not solely on weight loss, but on holistic, behavior-driven health outcomes in adolescent obesity care.

**Clinical trial registration:**

CTRI/2019/04/018,834.

## Graphical abstract

**Figure:**
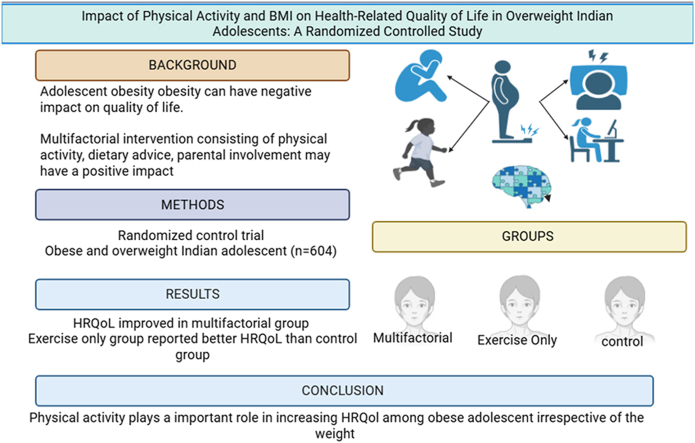

## 1 Introduction

The rising rates of overweight and obesity among adolescents have become a primary global health concern [1]. Overweight and obesity are associated with a broad spectrum of chronic diseases, including early-onset cardiovascular disorders, type 2 diabetes, metabolic syndrome, and respiratory conditions such as asthma [2]. Obesity can begin in adolescence and persist into adulthood, where it contributes to long-term health complications and a reduced quality of life [2].

In addition to the physical risks, adolescents with obesity frequently experience psychosocial challenges [3]. Adolescent obesity is commonly linked to low self-esteem, negative body image, depression, anxiety, and social isolation [4]. These psychological issues not only affect the mental and emotional health of adolescents but can also interfere with school performance, peer relationships, and family dynamics [4]. This combined psychosocial impact on adolescents and their caregivers burdens the healthcare system and society [4]. Several studies have found that adolescents with overweight or obesity tend to report lower health-related quality of life (HRQOL) than their normal-weight peers [[5], [6], [7], [8]]. HRQOL is a multidimensional concept encompassing physical, emotional, social, and school functioning [9]. It has emerged as a vital outcome measure in obesity research, providing insight into how chronic health conditions affect daily life and well-being [9]. HRQOL assessments have been increasingly used in clinical and public health research to evaluate treatment outcomes, identify disparities in healthcare, and guide resource allocation and program strategies [10,11]. Numerous factors influence adolescent HRQOL, including body weight, emotional health, and behavioral habits [12]. One study in Sri Lanka reported that children with higher BMI had notably lower HRQOL scores, particularly in physical, social, and school functioning domains, suggesting that increasing body weight can negatively impact multiple aspects of an adolescent's daily experience [13].

Physical activity (PA) is another factor consistently connected with better adolescent HRQOL [14]. Research has shown that adolescents who engage in moderate to vigorous PA report higher HRQOL, likely due to growth and fitness, greater muscle strength, reduced sedentary time, and enhanced mental well-being [[15], [16], [17], [18]]. A recent study suggested that PA may mediate the relationship between life satisfaction and HRQOL, highlighting its central role in adolescent development [19]. Although global studies support strong associations between BMI, PA, and HRQOL, these relationships are often shaped by cultural norms, family context, and social environments [[20], [21], [22], [23]]. Global findings may not be directly generalizable to Indian adolescents, who experience unique sociocultural pressures and school systems. Within India, most existing research on HRQOL has focused on adolescents with diagnosed co-morbidities [[24], [25], [26], [27], [28]], leaving a clear gap in understanding how BMI and PA affect HRQOL in otherwise healthy adolescents with overweight or obesity.

Given the rising rates of adolescent obesity in India, there is an urgent need for context-specific data. This study was designed to address that gap by exploring how changes in BMI and PA relate to overall and domain-specific HRQOL in Indian adolescents classified as overweight or obese. Findings from this research can inform the design of future programs that target not just weight reduction, but also improvements in quality of life and long-term health outcomes.

## 2 Methods

### 2.1 Study design and adolescents

This study is a secondary analysis of a randomized controlled trial that evaluated the impact of lifestyle interventions on PA, body mass index, and psychosocial outcomes among Indian adolescents classified in the higher weight category [29]. Full methodological details, including sample size calculation, randomization procedures, allocation concealment, intervention fidelity, and dropout handling, have been described in the primary trial publication (IEC: 536–2018) (CTRI/2019/04/018,834) [29].

Male and female students aged 11 to 16 were recruited from English-medium schools in Udupi, Karnataka, India. Adolescents were eligible if their body mass index (BMI) was above the 85th percentile for age and sex based on the Center for Disease Control and Prevention (CDC) growth charts [30]. This classification defines overweight as a BMI between the 85th and 95th percentile, and obesity as a BMI at or above the 95th percentile [30]. Adolescents were excluded if they had a diagnosis of obesity secondary to endocrine disorders or medications, were undergoing psychiatric treatment, could not commit to twice-weekly sessions during school hours, had any medical contraindication to PA, or were enrolled in other structured exercise programs. Adolescents who disclosed mental health concerns were referred to their school counselor, a general practitioner, or relevant community-based mental health services. Eligible individuals were randomly assigned to one of three groups: a Multifactorial program (MFI) group, an Exercise-only (EX) group, or a Control (CON) group. The MFI group received a comprehensive program that included structured PA sessions, lifestyle education, and parental involvement. The EX group received only the structured exercise component, while the CON group did not receive any strategy during the study period. Randomization was stratified by school and conducted using a computer-generated sequence. Allocation concealment was ensured using sealed opaque envelopes. The intervention lasted for 12 weeks, with bi-weekly sessions. Each session was conducted in schools during physical education classes, which lasted approximately 60 min. Each exercise session consisted of strength-based and aerobic activities tailored to adolescent capacity. Education sessions in the MFI group were conducted using a validated educational booklet and one-to-one sessions during parent-teacher meetings. Each education session addressed nutrition, behavior change, benefits of regular PA, and family dynamics. Follow-up assessments were completed at the starting point, post-program, 6 months, and 12 months. The intervention duration of 12 weeks was based on prior evidence supporting the effectiveness of short-term, school-based lifestyle programs for adolescents. This timeframe was also chosen to align with the academic calendar and minimize disruption to school routines. Ethical approval was obtained from the Institutional Ethics Committee of Kasturba Medical College and Hospital (IEC: 536–2018), and the study was registered with the Clinical Trials Registry of India (CTRI/2019/04/018,834).

### 2.2 Measurements

#### 2.2.1 Anthropometry

Height and weight were measured using standardized procedures and calibrated equipment, following guidelines based on the National Health and Nutrition Examination Survey protocols [31]. Height was measured using a stadiometer, and weight was recorded using digital weighing scales. Adolescents stood without shoes, with heels together and back straight, ensuring contact with the wall at the heels, buttocks, and head. BMI was calculated as weight in kilograms divided by the square of height in meters (kg/m^2^), and adolescents were classified using CDC BMI-for-age growth percentiles [31].

#### 2.2.2 Physical activity

PA levels were measured using the Physical Activity Questionnaire for Adolescents (PAQ-A), a validated self-report instrument designed to capture general activity over the past seven days [32]. The questionnaire includes items on PA during school hours, lunch breaks, after school, evenings, and weekends. Each question is scored on a 5-point Likert scale, with higher scores indicating greater activity levels [32]. The final PA score was derived by averaging the item scores.

#### 2.2.3 Health-related quality of life

HRQOL was measured using the Pediatric Quality of Life Inventory Version 4.0 (PedsQL 4.0), a widely used and validated tool for assessing quality of life in children and adolescents (Cronbach's alpha = 0.89) [33]. The PedsQL includes 23 items across four domains: physical functioning, emotional well-being, social functioning, and school performance. Items are rated on a 5-point Likert scale ranging from 0 (never) to 4 (almost always), which are then reverse-scored and transformed to a 0–100 scale [33]. Higher scores indicate better HRQOL. Total scores and domain-specific means were computed by averaging the transformed scores of completed items in each section. (33)

#### 2.2.4 Statistical analysis

All analyses were performed using Jamovi statistical software (version 2.3, The Jamovi Project, 2023). Descriptive statistics were used to summarize demographic, anthropometric, and HRQOL variables. The primary outcome variable was HRQOL, with PA and BMI as the primary predictors.

To account for the repeated measures design and within-subject variability, linear mixed-effects models (LMMs) were used. These models were also applied to assess each HRQOL domain individually (physical, emotional, social, and school functioning). The normality of residuals was checked using Q-Q plots, and model assumptions were satisfied. Separate models were run for each group (MFI, EX, and CON) to allow group-specific interpretation of the program effects over time.

## 3 Results

### 3.1 Participant characteristics

A total of 604 adolescents were enrolled, with 306 (50.7 %) male and 298 (49.3 %) female. Based on CDC BMI-for-age percentiles, 67 % of participants were classified as overweight, and 33 % were classified as being obesity. The distribution of these classifications was balanced across the three study groups with nearly equal representation across the three groups: CON (n = 208), EX (n = 199), and MFI (n = 197). The group distribution is outlined in Table 1.

**Table 1:** Participant distribution by group.

Group	Frequency	Percentage (%)
CON	208	34.4
EX ONLY	199	32.9
MFI	197	32.6

### 3.2 Trends over time: PA, BMI, and HRQOL

Throughout the study, the MFI group showed the most clear positive changes in health-related quality of life (HRQOL). On average, their HRQOL scores rose from approximately 44.6 at initial assessment to 69.0 at the final follow-up. PA levels also steadily improved from a mean score of 3.51–4.56. Interestingly, BMI grew modestly during the approach (from 23.13 to 25.79), suggesting the enhanced quality of life occurred independently of apparent weight loss. The EX group showed moderate gains, with HRQOL increasing from 46.5 to 54.2, and PA improving from 3.43 to 3.92. BMI in this group also grew (23.23–26.30), although to a lesser extent than in the CON group. In contrast, the CON group saw little change in HRQOL or PA, but experienced a consistent growth in BMI (22.99–27.17), indicating progression toward higher weight status without linked well-being benefits.

### 3.3 Predictors of overall HRQOL: mixed-effects model findings

Linear mixed-effects modeling highlighted apparent differences across the strategy arms. PA activity and BMI were positively associated with HRQOL in the Multifactorial group, even as BMI scores increased modestly over the study period. Each one-unit rise in PA was linked with an average 9.3-point gain in HRQOL (p < 0.001), while BMI was also positively related (β = 1.15, p < 0.001). The EX group showed similar trends, although the associations were weaker. PA was associated with a 4.47-point rise in HRQOL (p < 0.001), and BMI had a more negligible yet straightforward positive effect (β = 0.51, p < 0.001). Neither PA nor BMI showed a meaningful relationship with HRQOL in the CON group. These findings are presented in Table 2.

**Table 2:** Association of PA and BMI with HRQOL.

Group	PA (β)	PA (p-value)	BMI (β)	BMI (p-value)
MFI	9.3	<0.001	1.15	<0.001
EX ONLY	4.47	<0.001	0.51	<0.001
CON	−0.33	0.645	0.02	0.830

### 3.4 Effect of PA and BMI on HRQOL domains by group

The influence of PA and BMI on health-related quality of life (HRQOL) varied across program groups and HRQOL domains. The most consistent and clinically meaningful improvements were observed in the MFI group, followed by the EX group. In contrast, the CON group showed minimal to no clear associations.

### 3.5 Multifactorial program group

Adolescents in the MFI group, who received a combination of exercise, behavioral counseling, and lifestyle education, showed meaningful improvements in all HRQOL domains. Physical Functioning improved the most, with PA showing a strong association (β = 12.78, p < 0.001). This suggests that students felt more physically capable, experienced less fatigue, and were better able to participate in daily tasks and recreational activities. BMI was also positively linked with this domain (β = 1.87, p < 0.001). Emotional Functioning saw notable gains related to both PA (β = 10.46, p < 0.001) and BMI (β = 1.21, p < 0.001). These findings likely reflect mood, self-esteem, and stress management improvements—key psychosocial outcomes commonly affected by PA and structured support. Social Functioning improved substantially as well, with PA (β = 14.74, p < 0.001) and BMI (β = 1.70, p < 0.001) showing strong positive associations. School Functioning also benefited meaningfully, with higher PA (β = 9.79, p < 0.001) and BMI (β = 1.35, p < 0.001) linked to better focus, reduced absenteeism, and improved academic engagement. The multifaceted nature of the approach likely contributed to better emotional regulation and cognitive readiness for learning.

### 3.6 Exercise-only group

Adolescents in the EX group, who received only the structured PA component, showed moderate improvements across domains, though less consistently than the MFI group. Emotional Functioning was the domain most strongly influenced by the strategy, with meaningful positive associations for both PA (β = 11.53, p < 0.001) and BMI (β = 1.84, p < 0.001). Physical Functioning also improved (β = 5.36, p < 0.001 for PA; β = 0.49, p < 0.001 for BMI), though the effect sizes were smaller than those in the MFI group. School Functioning showed modest but statistically meaningful progress related to PA (β = 3.02, p = 0.034), with a marginal association for BMI (β = 0.43, p = 0.078). Social Functioning showed only a marginal relationship with PA (β = 2.43, p = 0.075) and no meaningful association with BMI.

### 3.7 CON group

The CON group, which received no intervention, showed minimal changes and largely unclear associations across domains. Emotional Functioning was the only domain to show a clear relationship, and only with BMI (β = 1.11, p < 0.001), possibly reflecting residual psychosocial adaptation in some students. Across Physical, Social, and School Functioning, no clear associations were observed between PA or BMI and HRQOL outcomes.

**Table:** 

Domain	Group	PA (β)	PA (p-value)	BMI (β)	BMI (p-value)
Physical Functioning	Multifactorial	12.78	<0.001	1.87	<0.001
Emotional Functioning		10.46	<0.001	1.21	<0.001
Social Functioning		14.74	<0.001	1.7	<0.001
School Functioning		9.79	<0.001	1.35	<0.001
Physical Functioning	Exercise-only	5.36	<0.001	0.49	<0.001
Emotional Functioning		11.53	<0.001	1.84	<0.001
Social Functioning		2.43	0.075	0.18	0.442
School Functioning		3.02	0.034	0.43	0.078
Physical Functioning	Control	1.03	0.373	0.11	0.497
Emotional Functioning		0.51	0.742	1.11	<0.001
Social Functioning		−0.66	0.659	−0.1	0.629
School Functioning		0.3	0.845	−0.32	0.129

## 4 Discussion

This study explored how PA and body mass index (BMI) interact over time to influence HRQOL among adolescents classified with higher weight and adolescents with obesity in India. Using a randomized controlled design with longitudinal follow-up, we observed that structured interventions, primarily those combining exercise, education, and behavioral support, were associated with substantial improvements in HRQOL across multiple domains. These findings underscore the importance of adopting holistic, multidimensional approaches to adolescent obesity management that extend beyond weight loss alone. These findings suggest that adolescent health outcomes should not be assessed solely through weight-centric metrics, but rather through a multidimensional lens that includes emotional and functional health.

The most notable benefits were observed in the multifactorial intervention group, which included structured PA, lifestyle education, and behavioral counseling. Adolescents in this group showed consistent improvements across all HRQOL domains: physical functioning, emotional well-being, social interaction, and school performance. These results are consistent with the understanding that adolescents face not only physical challenges related to overweight but also emotional and social vulnerabilities [34,35]. A program that combines physical training with psychosocial support may be more effective in addressing the complex realities of adolescent development, including self-esteem, peer comparison, and identity formation [36,37].

The exercise-only group also showed improvements in HRQOL, although the magnitude of gains was smaller. This confirms the known benefits of PA for adolescent well-being, including enhanced cardiovascular fitness, strength, sleep quality, and mood regulation [16,38,39]. Mechanistically, exercise modulates neurochemical pathways—including endorphins, dopamine, and serotonin—which play key roles in emotional stability and stress reduction. [[40], [41], [42]]. However, the absence of structured psychosocial or educational components may have limited broader improvements, particularly in social and school domains. One of the more unexpected findings was the positive association between BMI and HRQOL in both intervention groups. This contrasts with previous literature that has generally identified an inverse relationship, where higher BMI is associated with poorer HRQOL due to body dissatisfaction, social stigma, or functional limitations [[43], [44], [45], [46]]. Several possible explanations may help contextualize this finding. First, it is essential to recognize that BMI is a crude measure of adiposity, particularly in adolescents undergoing growth spurts or changes in body composition [47,48]. It is plausible that participants gained lean mass through the exercise intervention without significantly reducing total body mass, leading to an unchanged or even elevated BMI that does not reflect improved health status [49]. Second, adolescents participating in structured, supportive programs may have developed more positive self-perceptions, independent of weight loss [50]. Improvements in physical fitness, social interaction, and emotional coping may have contributed to greater confidence and self-acceptance, enhancing HRQOL [50]. This finding suggests that improvements in HRQOL may have occurred despite increases in BMI, rather than because of them. It is plausible that the structured intervention enhanced adolescents’ self-perceptions, emotional resilience, and social participation factors that can independently improve HRQOL [6]. The Emotional Functioning result suggested a strong mood-enhancing and confidence-building effect from consistent exercise alone. BMI may have become less psychologically salient in this context, especially if adolescents internalized a healthier narrative about their bodies. This interpretation aligns with recent calls to adopt weight-neutral or weight-inclusive approaches to obesity care in youth, focusing on health behaviors and functional capacity rather than BMI alone [51,52]. Nonetheless, this finding must be interpreted cautiously. Without direct assessment of body composition (e.g., fat mass vs. lean mass), pubertal stage, or psychological mediators such as body image and perceived stigma, it is difficult to explain why BMI was positively associated with HRQOL definitively. It is also possible that social desirability bias, particularly in self-reported questionnaires, may have influenced HRQOL responses [53]. Future studies should incorporate more sensitive tools such as dual-energy X-ray absorptiometry (DXA), qualitative interviews, or validated body image scales to investigate these pathways further. It was interesting to note that the physical Functioning improved, suggesting that while PA helped adolescents feel fitter, the absence of behavioral support may have limited broader improvements. Furthermore, the modest improvement in Social Functioning highlighted a possible cognitive benefit of PA even without additional strategy layers.

In contrast, the CON group, which received no structured intervention, showed no significant improvements in HRQOL. This reinforces the critical role of guided, supportive programs in facilitating adolescent behavior change. Without structured opportunities for PA or emotional support, adolescents may lack the resources or motivation to improve their health autonomously. This underscores the importance of structured approaches in supporting adolescents' physical and psychosocial well-being.

These findings echo the results of previous school-based interventions, such as the MEND and HEALTHY programs, which also reported improved psychosocial and functional outcomes in youth classified with higher weight, following structured, multifaceted interventions [54,55]. Our domain-specific analysis offers further insight into the varied effects of PA and BMI on adolescent functioning. Physical functioning consistently improved across intervention groups, reflecting fitness, strength, and coordination gains [56]. These changes are significant for Adolescents in the overweight category, who may initially experience limitations in mobility or endurance. Emotional functioning also improved, especially in the MFI group, suggesting that psychological support and behavior change strategies are effective in reducing stress, anxiety, and negative mood states. This is supported by work from Tsiros and colleagues, who noted emotional health as one of the most sensitive domains to change following pediatric obesity interventions [57].

Social functioning improved significantly in the MFI group, but less in the EX-only group. This may reflect the added value of peer support, group activities, and psychoeducation in building social confidence and reducing isolation [58]. Adolescents often struggle with body image concerns and peer dynamics, which cannot be fully addressed through PA alone [59]. The MFI approach may indicate that increased participation in group activities and better body confidence helped adolescents engage more comfortably in peer interactions. The EX-alone intervention results suggest that improvements in peer relationships may be limited without behavioral counseling or structured group interaction.

Similarly, school functioning was higher in the intervention groups, possibly due to improved sleep quality, energy levels, and emotional regulation, which influence concentration and academic engagement [60]. These findings support the need for a paradigm shift in adolescent obesity care from a weight-centric model to one that emphasizes health behavior, functional ability, and emotional well-being [52,61]. While BMI remains a valuable screening tool at the population level, it does not capture the complexity of adolescent health, particularly in intervention contexts [62]. Clinical programs should consider incorporating multidimensional outcome measures, including HRQOL, mental health, and fitness parameters, to reflect meaningful change better [63]. The 12-week intervention aligns with established school-based programs and was chosen for feasibility within academic schedules. Despite duration compactness, the 12-month follow-up enabled assessment of long-term outcomes, and findings suggest even short-term interventions can yield lasting HRQOL benefits. Notably, programs such as MEND and HEALTHY have demonstrated significant improvements in both psychosocial and physical outcomes with comparable intervention lengths [54,55]. Future studies may explore whether extended or repeated programs enhance BMI outcomes and behavior maintenance.

In the context of low- and middle-income countries like India, schools provide an accessible and cost-effective platform for delivering such integrated interventions [64]. Policymakers should consider embedding PA, mental health education, and nutritional guidance within the school curriculum, reaching adolescents at a formative stage [64]. An important observation from this study was the greater increase in BMI in the control group compared to both intervention groups. While all three groups showed a rise in BMI over the study period, the CON group exhibited the steepest trajectory, moving toward higher weight status without parallel gains in HRQOL. This divergence may suggest that the absence of structured intervention not only limited improvements in quality of life but also allowed for greater weight gain, potentially exacerbating psychosocial and physical challenges. Conversely, the structured programs in the MFI and EX groups may have mitigated excessive BMI increases through engagement in physical activity and lifestyle awareness, even if BMI did not decline. These findings emphasize the protective role of intervention strategies against unhealthy weight progression and highlight the importance of considering BMI changes as both an outcome and a potential modifier of HRQOL trajectories in adolescent obesity research.

Despite its strengths, including large sample size, longitudinal design, and statistical modeling, this study has limitations. PA was assessed using self-report measures, which may be prone to recall and social desirability bias. Future studies should include objective tools such as accelerometers to validate PA levels. BMI as the sole anthropometric marker limits insight into accurate body composition changes. Additionally, the sample was limited to school-going adolescents in an urban setting, which may affect the generalizability of findings to rural or out-of-school populations. Additionally, we did not assess cardiometabolic or physiological markers such as blood pressure, lipid profiles, insulin resistance, or inflammatory biomarkers. While these measures provide important insight into the biological effects of physical activity, our study focused on HRQOL as a pragmatic and scalable outcome in school settings. Future research should consider integrating both psychosocial and physiological metrics to provide a more comprehensive assessment of adolescent health. Our school-based intervention focused on behavioral strategies for otherwise healthy adolescents. We acknowledge that pharmacotherapy and bariatric surgery play important roles in managing severe pediatric obesity. These clinical approaches are essential in multidisciplinary care, and future research should explore how school-based programs can complement such strategies within a tiered care model.

## 5 Conclusion

In conclusion, this study adds to the growing body of evidence supporting the efficacy of structured, school-based programs in improving the quality of life of overweight and adolescents with obesity. The results highlight the need for multidimensional, behavior-focused strategies that promote weight loss, physical capability, emotional resilience, and social integration. Such approaches may offer a more sustainable and developmentally sensitive pathway to improving adolescent health.

## Conflict of interest

All authors declare that they have no known financial or personal relationships that could have appeared to influence the work reported in this paper.

Declare that they have no conflicts of interest relevant to the content of this manuscript.
